# Post-marketing assessment of content and efficacy of preservatives in artemisinin-derived antimalarial dry suspensions for paediatric use

**DOI:** 10.1186/1475-2875-6-12

**Published:** 2007-01-26

**Authors:** Magnus A Atemnkeng, Katelijne De Cock, Jacqueline Plaizier-Vercammen

**Affiliations:** 1 Department of Pharmaceutical Technology and Physical Pharmacy, Faculty of Medicine and Pharmacy, Vrije Universiteit Brussel, Laarbeeklaan 103, B-1090 Brussels. Belgium

## Abstract

**Background:**

Artemisinin-derivative formulations are now widely used to treat falciparum malaria. However, the dry powder suspensions developed for children are few and/or are of poor quality. In addition to the active compound, the presence of a suitable preservative in these medicines is essential. In this study, an evaluation of the preservative content and efficacy in some dry suspensions available on the Kenyan market was performed.

**Method:**

UV spectrophotometry was used to identify the preservatives in each sample while HPLC-UV was used for quantification. After reconstitution of the powders in water, the dissolution of the preservatives was followed for 7 days. Antimicrobial efficacy of the preservatives was assessed by conducting a preservative efficacy test (PET) following the European pharmacopoeia standards.

**Results:**

Four different preservatives were identified namely methylparahydroxybenzoate (MP), propylparahydroxybenzoate (PP), benzoic acid and sorbic acid. MP and PP were identified in Artesiane^® ^(artemether 300 mg/100 ml), Alaxin^® ^(dihydroartemisinin 160 mg/80 ml) andGvither ^® ^(artemether 300 mg/100 ml) respectively. Sorbic acid was presentin Artenam^® ^(artemether 180 mg/60 ml) while benzoic acid was identified in Santecxin^® ^(dihydroartemisinin 160 mg/80 ml) andArtexin^® ^(dihydroartemisinin 160 mg/80 ml) respectively. Cotecxin^® ^(dihydroartemisinin 160 mg/80 ml) did not contain any of the above preservatives. After reconstitution in water, preservativesin 50%(3/6) of the products did not completely dissolve and the PET results revealed that only Artenam^® ^and Gvither^® ^met the requirements for antimicrobial efficacy. The other products did not conform.

**Conclusion:**

These results show that paediatric antimalarial dry powder formulations on the market may contain ineffective or incorrect amounts of preservatives. This is a potential risk to the patient. Studies conducted on the dry powder suspensions should include the analysis of both the active ingredient and the preservative, including the efficacy of the latter.

## Background

The artemisinin-derivatives, artemether, artesunate, arteether and dihydroartemisinin, are currently the most potent antimalarial medicines on the market. They are widely available in the different pharmaceutical dosage forms including tablets, injections, suppositories and dry powders [1].

Since artemisinin and its derivatives are poorly water-soluble and are not very stable in solution, the preparations have to be formulated in the dry form for subsequent reconstitution into a wet suspension with water just before use. The dry powders for reconstitution are normally designed for children from 0–5 years of age, who are not able to swallow tablets. In the malaria endemic countries, living conditions are often poor, including scarce access to clean portable drinking water [2]. As a result, microorganisms can easily thrive when the dry powder is reconstituted with poor quality water. In addition, children suffering from malaria, as well as AIDS or typhoid, have a weakened immunological system and are, therefore, more susceptible to other infections. Moreover, the drugs are packaged in multiple dose containers, making the preparation highly susceptible to contamination following frequent use. Hence, pharmaceutical preparations which need an aqueous vehicle such as syrups and powders for oral suspensions require safeguards from microbial contamination, which may affect product stability or infect the consumer. This is accomplished by the addition of antimicrobial agents in the formulation to destroy and inhibit the growth of those organisms that may contaminate the product during manufacture or use [3].

The International Committee on Harmonization (ICH) guidelines [4] requests that for submission of drug registration dossier on dry powders for oral suspensions, data should be provided for the content of the active pharmaceutical ingredient (API) as well as the type(s) and amount(s) of the preservative(s) used. In addition, the efficacy of the antimicrobial preservation should be demonstrated by challenging the reconstituted suspension in its final container with specified microorganisms. This implies that the preservative used in the dry powder must completely dissolve on addition of water to impart the preservation action.

Sources of this microbial contamination may include air and water, manufacturing equipment, manufacturing personnel and/or the consumer [5]. Bacterial contamination of products through consumer use, has resulted in presence of mixed and harmful microbial flora in the product [6].

Major studies on antimalarial formulations are limited to the active ingredients without mention of the preservatives when studied in syrups and dry powders. In view of the biological role that this excipient plays towards the maintenance of the preparation and the recovery of the patient, there is a dire need for greater attention and awareness directed towards the importance of preservation in paediatric formulations.

Several chemical preservative agents exist and have been widely employed in the cosmetic, food and pharmaceutical industries [5]. For oral use, the choices of the preservatives are limited. These include benzoic acid (BA) C_6_H_5_COOH and sorbic acid (SA) C_5_H_7_COOH, which are generally effective to control mould and yeast growth, and the parahydroxybenzoic acid esters: methylparaben (MP) C_6_H_4_(OH)COOCH_3 _and propylparaben (PP) C_6_H_4_(OH)COOC_3_H_7, _which are most commonly used to control bacterial growth due to their broad antimicrobial spectrum with good stability and non-volatility [7]. MP and PP are usually used in combination as they possess a synergistic activity when used together. However, overuse of preservatives may cause allergic reactions hence, they should be shown not to be cytotoxic or sensitizing [8,9].

Recently, the artemisinin-derivative drugs have become a major target for counterfeiters. Fake and substandard versions of original brands have previously been reported in Southeast Asia [10,11] and now in Africa [12]. The substandard copies were present in all dosage forms but most especially in the tablets and dry powders. In the latter, quality analysis should also be performed on the preservatives. No report has been published on efficacy of preservatives in artemisinin-like antimalarial drugs.

Thus, the aim of this study was (1) to identify the commonly used antimicrobial agents in the artemisinin-containing dry suspensions on the market, (2) study the dissolution profiles of these preservatives after reconstituting in water, (3) evaluate the activity of the preservatives by performing the preservative efficacy test (PET) on the wet suspension and (4) describe some simple analytical procedures for these analytes in dry powders. The different high performance liquid chromatography (HPLC) methods used were validated for each analyte.

## Methods

### Materials and reagents

Potassium dihydrogenphosphate and sodium hydroxide (both Ph. Eur. grade) were obtained from Merck (Darmstadt, Germany) and HPLC grade methanol and acetonitrile were supplied by Fisher Scientific (Leicestershire, UK). Glacial acetic acid was obtained from JT Baker (Deventer, The Netherlands) while ammonia (pro analysi) was supplied by Merck (Darmstadt, Germany). Methylparaben and propylparaben were obtained from Federa (Brussels, Belgium), while benzoic acid (pro analysi) was supplied by Merck (Darmstadt, Germany). Sorbic acid was bought from Certa (Braine l'Alleud, Belgium). De-ionized milli-Q water was used throughout the experiment.

### Thin layer chromatography (TLC) for the identification of preservatives

Of the seven dry suspensions, only the Artenam^® ^and Artesiane^® ^samples indicated the type of preservative(s) used on the package insert. From the literature, the commonly used preservatives in oral aqueous pharmaceuticals were retrieved and used to identify the preservatives in the other samples. The TLC procedure described in the Ph. Eur. IV for the identification of parabens was initially tested to separate the four preservatives.

Ca. 100 mg of each of the reference preservative powder was weighed in separate 100-ml flasks and dissolved to the mark with methanol. The stationary phase was 10 cm × 20 cm RP-18 F_254S _silica gel plates from Merck (Darmstadt, Germany). The initial eluent was composed of 70 volumes of methanol, 30 volumes of water and 1 volume of glacial acetic acid. Several other compositions were tested to efficiently separate the four components on a single plate (see Table 1). Five μl of each standard solution was manually spotted using a glass capillary pipette at 2 cm spot distance. The plates were then developed in Camag^® ^TLC tanks presaturated with mobile phase. Development time was dependent on eluent composition, but ± 30 min was sufficient for most. The plates were allowed to dry in a well ventilated room. Visualization was on UV at 254 nm with a Camag^® ^Universal UV Lamp (Muttenz, Switzerland).

**Table 1:** Mobile phase compositions for the separation of preservatives by TLC (detected at 254 nm)

	Retardation factor (R_F_)			
Mobile phase (v/v/v)	Methylparaben	Propylparaben	Sorbic acid	Benzoic acid

CH_3_OH/H_2_O (80/20)	0.70	0.57	0.68	0.69 (Faint spot)
CH_3_OH/H_2_O/CH_3_COOH (70/30/1)	0.47	0.28	0.47	0.50 (Faint spot)
CH_3_OH/H_2_O/CH_3_COOH (80/20/1)	0.75	0.67	0.75	0.75
CH_3_OH/H_2_O/CH_3_COOH (80/20/3)	0.57	0.48	0.64	0.64 (Faint spot)
CH_3_OH/H_2_O/NH_3 _(80/20/1)	0.80	0.70	0.87	Highly faint spot
CH_3_CN/H_2_O/CH_3_COOH (95/5/1)	0.88	0.81	0.84	Highly faint spot

### UV spectrophotometry

To identify the preservatives in the other suspensions, methanol was added to each powder bottle, mixed and centrifuged at 3,000 rpm (*g *= 1,512) for 15 min. The supernatant was collected and appropriate dilutions were made in methanol. Spectra acquisition of the samples was done against a standard solution on a Uvikon 860 spectrophotometer (Kontron Instruments, Massachusetts, USA) connected to a Plotter 800 Integrator (Kontron Instruments, Massachussetts, USA). Pure methanol was used as the blank.

### HPLC instrumentation

The chromatographic system for the preservatives (MP, PP, BA and SA) consisted of a Merck-Hitachi L-6000 pump, a Perkin-Elmer LC 90 UV spectrophotometric detector connected to a Merck-Hitachi D-2500 Chromato-Integrator. The stationary phase in each case was a reversed-phase Nucleosil^® ^120-4 C_18 _column, 125 mm long by 4 mm (i.d) and 5 μm particle size from Macherey-Nagel (Düren, Germany) except for sorbic acid where a Lichrospher^®^250 × 4 mm, 5 μm particle size column from Merck(Darmstadt, Germany)was used. The eluent for the parabens consisted of an acetonitrile : KH_2_PO_4_(0.05 M, pH 5.0) buffer (300:700, v/v) mixture. The mobile phase of sorbic acid was composed of a mixture of acetonitrile : water : KH_2_PO_4_(0.05 M, pH5.0) buffer,(100:690:240, v/v/v)and benzoic acid was separated using acetonitrile :KH_2_PO_4_(0.05 M, pH 5.0) buffer(100:900, v/v) mixture. Detection of MP and PP was achieved on UV at 254 nm, 290 nm for sorbic acid and 226 nm for benzoic acid. All analyses were done isocratically at a flow rate of 1.0 ml/min and 20 μl of each sample was injected. In all experiments, the buffer was adjusted to there quired pH with sodium hydroxide and filtered using a 0.45 μm pore size membrane filter before use.

### Standard solutions preparation

A bulk powder mixture comprising of ca. 0.08% MP and 0.02% PP was prepared and from this mixture about 50 mg was accurately weighed in a 50-ml flask. This was completely dissolved to the mark with pure methanol. The solution was then diluted (100x) with the same solvent for analysis. Sorbic acid standard solution was prepared by weighing ca. 160 mg of it and dissolving in 50 ml methanol. Appropriate dilutions were then made, first 10x in methanol : water (4 : 1, v/v) mixture and then 2.5x with the mobile phase for injection. Benzoic acid standard was prepared by accurately weighing 60 mg of it in a 50-ml volumetric flask and dissolving to the mark with a methanol : water (900 : 100, v/v) mixture. From this a 20x dilution was made with a methanol : KH_2_PO_4 _(0.05 M, pH 5.0) buffer (50:50 v/v).

### Preservative content in dry powders

All powders analysed in this study were anonymously obtained from pharmacies within Nairobi in Kenya (East Africa). An Artenam^® ^semi-industrial batch dry powder suspension containing artemether (180 mg/60 ml) was added to the study. From each product the following were noted: type and dose of active ingredient and type of preservative (if indicated) and registration status. All analyses were performed before the expiry dates of the product.

Powder in each bottle was shaken to free the particles. For the dihydroartemisinin dry powders, exactly 200 ml of methanol : water (80 : 20, v/v) mixture was added to reduce the influence of the matrix and powder volume on the analysis. This solvent mixture was necessary to dissolve both the active and the preservative in order to use the content of the same bottle for both analyses. For the artemether dry powders, exactly 200 ml of pure methanol was added to the content. All the bottles were then thoroughly mixed and left on the shaking apparatus for at least 1 hour followed by ultrasonication for 15 min. Part of the suspension was transferred to 5-ml Falcon^®^tubes and centrifuged at 3,000 rpm (*g *= 1,512) for 15 min.

### Dissolution of preservatives in the reconstituted suspensions

The instructions described by each manufacturer were followed for reconstitution. Milli-Q water was added to each powder, well mixed till complete dispersion and part of the suspension was taken to determine its pH. From the rest a suitable volume was transferred to Falcon^® ^tubes and centrifuged at 3,000 rpm (*g *= 1,512) for 15 min. For the more viscous suspensions, the centrifugation step was repeated on the supernatant. The density of the supernatant was measured and subsequent volumes were determined by sample weighing. Appropriate dilutions of the supernatant were done for HPLC analysis at the following time points: immediately after reconstitution (t_0_), 6 hours, 24 hours, 4 days and 7 days respectively; the maximum period necessary for a complete treatment of severe malaria and during which the suspension is supposed to be stable.

### Preservative efficacy test

The method described in the European Pharmacopoeia IV 5.1.3 'Efficacy of Antimicrobial Preservation' was used [3]. The test consisted of challenging the reconstituted suspensions in their final containers with a prescribed inoculum of the following micro-organisms: *Pseudomonas aeruginosa *ATCC 9027, *Staphylococcus aureus *ATCC 6538, *Escherichia coli *ATCC 8739, *Candida albicans *ATCC 10231, *Zygosaccharomyces rouxii *NCYC 381 and *Aspergillus niger *ATCC 16404. The inoculated preparations were then stored at ambient temperature and samples were withdrawn at specified time intervals and the remaining micro-organisms counted.

## Results

### Preservative identification

**Figure 1:**
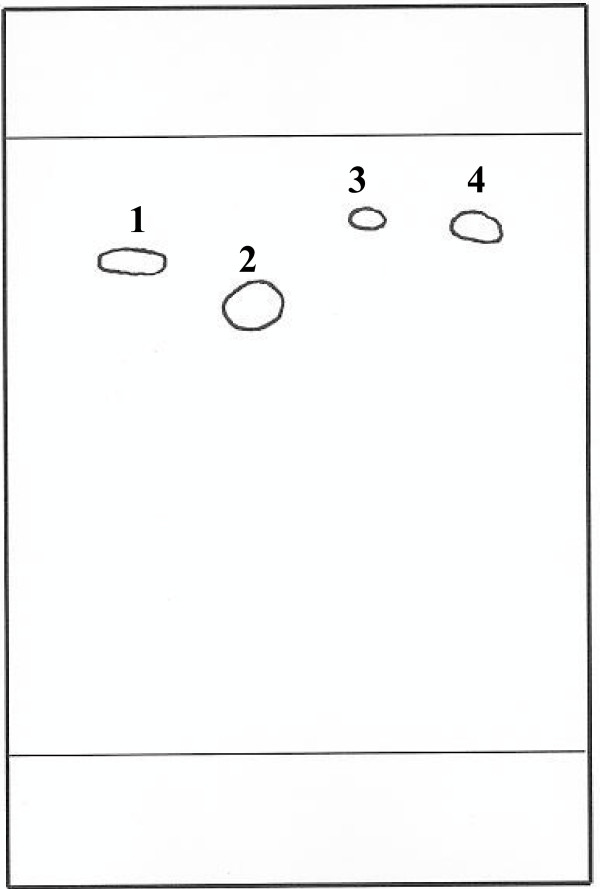
TLC plate showing the separation of preservatives (1 = methylparaben, 2 = propylparaben, 3 = benzoic acid, 4 = sorbic acid) using a solvent mixture of methanol : water : ammonia (80 : 20 : 1, v/v/v)

TLC experiments were done using the standard solutions of methylparaben, propylparaben, benzoic acid and sorbic acid to rapidly check the possibility of separating and identifying all four preservatives on a single plate. Visualization was done at 254 nm. The spots of MP, PP and SA were clearly visible on the plates. Only benzoic acid showed faint spots. Methylparaben and propylparaben were well separated from each other but there was more or less a constant R_F_(retardation factor) value for methylparaben and sorbic acid when different solvent systems were tried (Table 1). Changing eluent compositions did not effectively resolve all four analytes. A system that came close to a good separation was methanol : water : ammonia (80 : 10 : 1, v/v/v) with R_F_values of 0.80, 0.70, 0.87 for MP, PP and SA respectively (Figure 1). The manufacturers and origin of the different powders are presented in Table 2. UV spectra revealed the presence of four different preservative in the dry powders; MP and PP in Artesiane^®^, Gvither^® ^and Alaxin^®^, benzoic acid in Artexin^® ^and Santecxin^® ^and sorbic acid in Artenam^® ^respectively. Cotecxin^® ^did not exhibit any clear UV spectrum however, a personal correspondence with the manufacturers stated chlorbutanol as the preservative used. For quantification, spectrophotometry was not a good method since preservatives that exist in combination such as the parabens will absorb at the same wavelength [13]. In addition, an excipient that can interfere with the analyte cannot be separated on UV thus, HPLC-UV was used in subsequent experiments.

**Table 2:** Concentration of preservative(s) in dry suspensions after reconstitution

Product/Manufacturer	Active ingredient/Dose	Preservative(s) identified	% preservative found	Normal value in H_2_O^+ ^(%)
Artenam^®^* Arenco, Belgium	Artemether 180 mg/60 ml	Sorbic acid	0.27	0.20
Gvither^® ^GVS Labs, India	Artemether 300 mg/100 ml	Methylparaben	0.18	0.08
		Propylparaben	0.06	0.02
Artesiane^® ^Dafra, Belgium	Artemether 300 mg/100 ml	Methylparaben	0.08	0.08
		Propylparaben	0.02	0.02
Alaxin^® ^GVS Labs, India	Dihydroartemisinin 160 mg/80 ml	Methylparaben	0.09	0.08
		Propylparaben	0.01	0.02
Artexin^® ^Sphinx Pharma, Kenya	Dihydroartemisinin 160 mg/80 ml	Benzoic acid	0.15	0.10
Santecxin^® ^Shsj, China	Dihydroartemisinin 160 mg/80 ml	Benzoic acid	0.03	0.10
Cotecxin^®^, Jiaxing Nanhu Pharma, China	Dihydroartemisinin 160 mg/80 ml	Chlorbutanol?	Not tested	0.50

* Semi-industrial batch, ^+ ^Source: Martindale (The Complete Drug Reference) 1999.

### Preservatives content and dissolution

In the dry powders, 0.076% MP and 0.020% PP were found in Artesiane^®^, 0.088% MP and 0.011% PP in Alaxin^® ^while 0.178% MP and 0.057% PP were found in Gvither^® ^respectively. 0.268% sorbic acid was present in Artenam^® ^while Santecxin^® ^contained 0.031% benzoic acid and Artexin^® ^0.148% benzoic acid (Table 2). The normal aqua concentrations of parabens used in pharmaceutical products are 0.08% MP and 0.02% PP (when used in combination), 0.10% BA and 0.20% SA respectively [14]. However, in a powder mixture with a complex matrix these amounts may vary.

**Figure 2:**
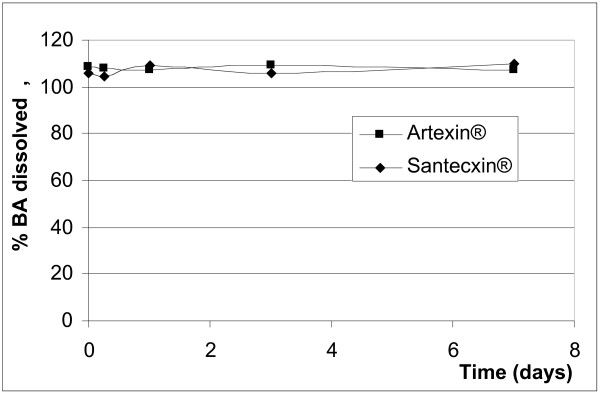
Dissolution profiles of benzoic acid (BA)

**Figure 3:**
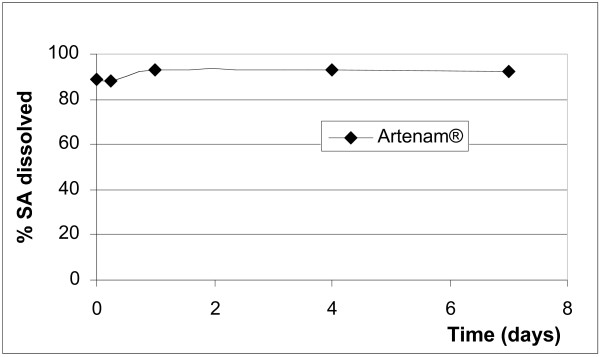
Dissolution profile of sorbic acid (SA)

**Figure 4:**
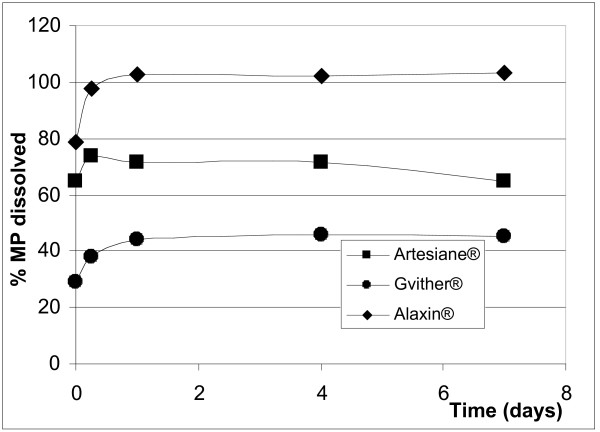
Dissolution profiles of methylparaben (MP)

**Figure 5:**
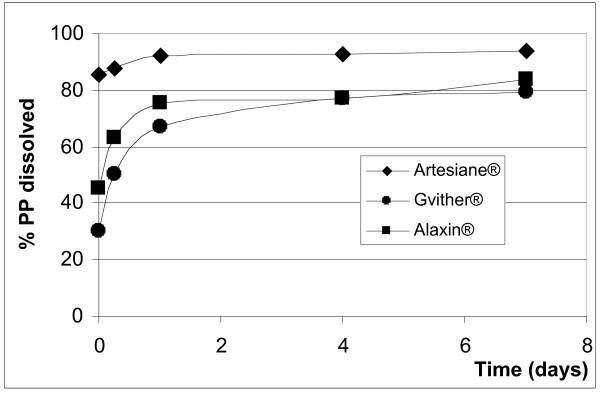
Dissolution profiles of propylparaben (PP)

After reconstitution (addition of water) only the benzoic acid (Santecxin^® ^and Artexin^®^) and the sorbic acid (Artenam^®^) containing products completely and immediately dissolved their preservatives (Figures 2 and 3) and the levels remained unchanged during the 7 days study period. None of the parabens immediately dissolved and the rate of dissolution differed between MP and PP in the same suspension and within different suspensions (Figures 4 and 5). Artesiane^® ^reached the 90% dissolution rate only after 24 hrs in PP and this remained stable for up to day 7. On the other hand, the total level of MP dissolved in the same sample did not exceed 74%. At t_0 _the amounts of preservative dissolved were 64.9% MP and 85.3% PP for Artesiane^®^, 28.8% MP and 30.1% PP for Gvither^®^, and 78.8% MP and 45.3% PP for Alaxin^®^respectively. Gvither^® ^possessed the most slowly dissolving parabens and only a maximum of 45.4% MP and 79.2% PP were present after 7 days (Figures 4 and 5). All the drug formulations were registered at the Drug Regulatory Agency of Kenya.

### Efficacy of antimicrobial preservation

A preservative efficacy test gives an indication of the antimicrobial activity of a preservative in a preparation. The preservative properties of the preparation were considered adequate if, in the conditions of the test, there was a significant fall or no increase in the number of micro-organisms at the conditions tested. In the tested wet suspensions only two samples, Artenam^® ^and Gvither^® ^met the specific requirements of the European Pharmacopoeia of PET in the killing of the micro-organisms. The other five products failed the test (Table 3). In the latter, different pathogen strains were observed with Santecxin^® ^and Cotecxin^® ^retaining the most species of microbes (≥ 4 out of 6 tested pathogens remained positive). Alaxin^® ^and Artexin^® ^were positive for two microbial species while Artesiane^® ^was positive for one. In all samples, the fungus *Aspergillus niger *was the most positively tested microbe.

**Table 3:** Efficacy of antimicrobial preservation of the artemisinin-derivative reconstituted suspensions

	pH wet suspension	Preservative	P. aeruginosa	S. aureus	E. coli	C. albicans	Z. rouxii	A. niger	Ph. Eur. requirements
Artenam^®^	4.50	Sorbic acid	-	-	-	-	-	-	Conforms
Gvither^®^	4.30	MP/PP	-	-	-	-	-	-	Conforms
Artesiane^®^	5.43	MP/PP	-	-	-	-	-	+	Does not conform
Alaxin^®^	4.40	MP/PP	-	+	-	-	-	+	Does not conform
Artexin^®^	5.90	Benzoic acid	+	-	+	-	-	-	Does not conform
Santecxin^®^	5.55	Benzoic acid	+	-	+	+	+	+	Does not conform
Cotecxin^®^	not tested	Chlorbutanol?	-	-	+	+	+	+	Does not conform

+ = positive for the tested microorganisms, - = negative for the tested microorganisms

MP = methylparaben, PP = propylparaben

### pH of reconstituted suspensions

Since a suitable pH is inevitable for the proper functioning of the preservatives, the pH of each reconstituted suspension was measured. The pH ranged from 4.50 to 5.90 in all wet suspensions (Table 3). The pH of two suspensions (Santecxin^® ^pH 5.55 and Artexin^® ^pH 5.90) exceeded by far the pK_a _of their preservative, benzoicacid (pK_a_4.20). The pH of Artenam^® ^wet suspension was 4.50(pK_a _sorbic acid 4.76) while the pH of the Artesiane^®^, Alaxin^® ^and Gvither^® ^formulations were respectively 5.43, 4.40 and 4.30(pK_a _parabens 8.4).

## Discussion

Artemisinin and its semi-synthetic derivatives are currently the most effective antimalarial compounds on the market. The dry suspension preparations of these drugs are of particular importance since they are specifically made for children (though the dose can also be adapted to an adult patient). This is a very vulnerable age group and more precaution is, therefore, necessary in formulating their medicines. In all such preparations a suitable preservative has to be added. In the tropics, where temperatures tend to be high in addition to high relative humidity, microbial contamination of the reconstituted suspension (and possible patient co-infection) can be common. In fact, in view of the possibility of using contaminated drinking water, most of the drug manufacturers advised that only boiled and cooled water should be used to reconstitute the suspension.

Regulatory law requires that preservatives must be listed by their common or usual names on ingredient labels of all drugs that contain them. Most manufacturers failed to indicate the type of preservative and the composition of other excipients in the formulation. This practice shades vital drug information, which is necessary for the patient, medical practitioners, researchers and the regulatory authorities.

It was not possible to use TLC alone to identify the preservatives present in the preparations due to the difficulty in separating methylparaben from sorbic acid as their R_F_values were nearly always the same (Table 1). With normal phase plates similar separation problems were encountered. The UV lamp used in spot visualization was set at two standard wavelengths only, 254 nm and 366 nm hence, the faint spots observed with benzoic acid would probably be due to its low absorption at 254 nm (λ_max _BA = 226 nm).

It is unclear what the recommended concentration of a preservative in a dry suspension is supposed to be. Nothing is mentioned in the United States Pharmacopoeia (USP) or the European Pharmacopoeia (Ph. Eur.) on the actual limits necessary hence, this leaves room for the formulators to employ different amounts of the same preservative; sometimes to detriment of the patient. This disparity in concentration is clearly observed in all the products (Table 2). The total amount of a preservative present in a dry powder is required to be available in the wet suspension. None of the paraben formulations met this criterion. In some drugs, values containing as low as 30% only of preservative were released after reconstitution. This leaves the drug susceptible to contamination. The ICH recommends that content limits of the preservative of between 90–110% at release should be acceptable. However, Ofner III et al [15] suggested that degradation of the preservative is acceptable as long as sufficient preservative is present to maintain effectiveness. To accomplish this, the use of the right type and quality of the preservative is primordial. For instance, esters of parahydroxybenzoic acids are slightly soluble in water and there is the danger that in the dry powders they may not dissolve fast enough after adding water. For such preparations, their acid salts such as sodium alkylparabens are preferred. Because the parabens took several days to reach their end concentration in the wet suspension, this may suggest that only the acid form of the preservative was used. Secondly, 100% of the dissolved preservative could not be retrieved due to the possibility of preservative adsorption on the solids and/or complex formation on the macromolecules in the suspension such as suspending agents [16]. Studies have shown that parabens adsorb to the surface of the container-closure system especially plastic containers. The latter are commonly employed in paediatric formulations [17]. All products used this packaging except the Artexin^® ^preparation which employed a brown bottle packaging. Due to the possibility of the interactions mentioned above, only the free fraction of the preservative can be active. Thus, the formulator has to be able to strike a balance between a level high enough to pass the preservative efficacy test and low enough to prevent adverse reactions.

The efficacy of a preservative depends not only on its concentration but also on the pH of the suspension. For preservatives that are carboxylic acids, only the un-ionized species is microbicidal. The pK_a _of such preservatives therefore determines the pH range in which the preservative is effective. The antimicrobial activity of the parabens, benzoic acid, sorbic acid and others certainly decreases as pH increases past their respective pK_a _[5]. It is, therefore, possible that though Artexin^® ^contained an high amount of benzoic acid (0.148%), its antimicrobial efficacy was not adequate since the pH of the reconstituted suspension of 5.90 exceeded the pK_a _of BA. This high pH dissociates the acid into the salt form leaving only a small undissociated fraction. On the other hand, Santecxin^® ^contained a very low amount of benzoic acid (only 0.031%) which probably was insufficient to impart the preservative's activity. The pH of all paraben formulations (Artesiane^®^, Gvither^® ^and Alaxin^®^) was lower than their pK_a _; hence inadequate antimicrobial efficiency could not be due to chemical dissociation. Though the Gvither^® ^sample contained the least dissolved parabens, its preservative efficacy test passed the requirements. This is probably due to the very high amounts of these substances present in the original formulation (0.178% MP and 0.057% PP respectively).

Though there are not many antimalarial dry powders on the market, a more prospective and large scale study involving samples collected in different endemic regions is necessary to ascertain the impact of preservation on the products. This initial study portrays the importance of preservation in aqueous antimalarial compounds.

## Conclusion

The high number of failures of the artemisinin-derivatives dry suspensions with respect to their antimicrobial preservation suggests that the surveillance of the marketed drugs may be ineffective in Kenya.

Effective preservation of paediatric formulations developed in multi-dose containers is necessary, as it contributes to the microbiological stability of the suspension as well as safeguard patient infection due to the formulation. Especially for children, paediatric medicines requiring a water phase need strict control on the content and efficacy of both the active ingredient as well as the preservative prior to registration. Above all, monitoring should continue after the drugs are on the market.

## Authors' contributions

MAA participated in the study design, analysis and interpretation of data and wrote the manuscript

KDC participated in the conception, experimental analysis of the study and revision of the manuscript

JPV participated in the overall supervision of the study and critically revised the content of the paper.
